# Exploring the Bioactive Potential of *Taraxacum officinale* F.H. Wigg Aerial Parts on MDA Breast Cancer Cells: Insights into Phytochemical Composition, Antioxidant Efficacy, and Gelatinase Inhibition within 3D Cellular Models

**DOI:** 10.3390/plants13192829

**Published:** 2024-10-09

**Authors:** Valentina Laghezza Masci, Elisa Ovidi, William Tomassi, Daniela De Vita, Stefania Garzoli

**Affiliations:** 1 Department for Innovation in Biological, Agro-Food and Forest Systems (DIBAF), University of Tuscia, Largo dell’Università, 01100 Viterbo, Italy; laghezzamasci@unitus.it (V.L.M.); eovidi@unitus.it (E.O.); william.tomassi@outlook.com (W.T.); 2 Dipartimento di Biologia Ambientale, Università di Roma “La Sapienza”, Piazzale Aldo Moro 5, 00185 Rome, Italy; daniela.devita@uniroma1.it; 3 Department of Chemistry and Technologies of Drug, Sapienza University, Piazzale Aldo Moro 5, 00185 Rome, Italy

**Keywords:** *Taraxacum officinale*, chemical analyses, antioxidant activity, matrix metalloproteinase, MDA cell line, 3D cell system

## Abstract

In this work, aerial parts of *Taraxacum officinale* F.H. Wigg. produced in Umbria, Italy, were chemically investigated by solid-phase microextraction/gas chromatography–mass spectrometry (SPME/GC-MS) to describe their volatile profile. The results obtained showed the preponderant presence of monoterpenes, with limonene and 1,8-cineole as the main components. Further analyses by GC/MS after derivatization reaction were performed to characterize the non-volatile fraction highlighting the presence of fatty acids and di- and triterpenic compounds. *T. officinale* methanol and dichloromethane extracts, first analyzed by HRGC/MS, were investigated to evaluate the antioxidant activity, cytotoxicity, and antiproliferative properties of MDA cells on the breast cancer cell line and MCF 10A normal epithelial cells as well as the antioxidant activity by colorimetric assays. The impact on matrix metalloproteinases MMP-9 and MMP-2 was also explored in 3D cell systems to investigate the extracts’ efficacy in reducing cell invasiveness. The extracts tested showed no cytotoxic activity with EC50 > 250 µg/mL on both cell lines. The DPPH assay revealed higher antioxidant activity in the MeOH extract compared with the DCM extract, while the FRAP assay showed a contrasting result, with the DCM extract exhibiting slightly greater antioxidant capacity. After treatment for 24 h with a non-cytotoxic concentration of 500 µg/mL of the tested extracts, gelatin zymography and Western blot analyses demonstrated that both MeOH and DCM extracts influenced the expression of MMP-9 and MMP-2 in MDA cells within the 3D cell model, leading to a significant decrease in the levels of these gelatinases, which are crucial markers of tumor invasiveness.

## 1. Introduction

The common Dandelion (genus *Taraxacum* F.H. Wigg), is a perennial plant belonging to the Asteraceae family. It grows well in temperate regions throughout the world and has a rather complex classification as it includes over three hundred species [1]. It has been used in traditional medicine and folk remedies to treat various diseases thanks to its therapeutic potential [2]. Its applications in the medical field are many as it can exert numerous effects such as antioxidant and free radical scavenging, anti-inflammatory and analgesic effects, hepatoprotective and hepatoregenerative effects, anti-diabetic, hypoglycemic properties, immunomodulatory effects, and neuroprotective activity [3]. More recent studies have also highlighted the use of *T. officinale* in the food sector [4].

*T. officinale* is a well-known plant with a variety of biological properties due to its richness in bioactive compounds [5]. Numerous types of preparations are produced by different methods (dried matrix, infusion, or extraction) and from different parts of the plant (leaves, flowers, and roots) [5].

The antioxidant activity is an essential process within the body to counteract or eliminate free radicals or to inhibit the formation of ROS. They can also impede lipid peroxidation, DNA damage and protein modification. Furthermore, antioxidants can enhance the activity of naturally occurring antioxidant enzymes such as superoxide dismutase (SOD), catalase, and glutathione peroxidase [6]. Natural antioxidants in food and medicinal plants can contribute to counteract oxidative stress and mitigate the effects on human health [7].

The production of reactive oxygen species (ROS) in the body can have both beneficial and physiological effects when present at low concentrations, and harmful or toxic effects when present at high concentrations. At low concentrations, they are involved in processes such as redox regulation, mitogenic responses, cellular signaling pathways or immune response functions, while at high concentrations, they can generate oxidative stress [8]. The oxidative stress plays a role not only in the toxicity of xenobiotics but also in various aspects of pathophysiology such as ischemia-reperfusion injury, vascular endothelium issues, deep injuries, organ dysfunction, shock, inflammation, sepsis, diabetic retinopathy, cognitive dysfunction, cataracts, heart disease, and cancer [9].

The aqueous fermented extract of *T. officinale* demonstrated significant antitumorigenic effects across various cancer cell lines, inducing apoptosis, inhibiting cell viability and migration, and disrupting mitochondrial integrity, while showing differential effects on non-tumorigenic cells. Different extracts and fractions of *T. officinale* showed cytotoxic and antiproliferative activities against a range of cancer cell lines, with varying mechanisms such as the induction of apoptosis, cell cycle arrest, and modulation of gene expression, suggesting the potential of *T. officinale* as a source for novel anticancer compounds that warrant further exploration through fractionation and compound isolation [10]. The antitumor activity observed on triple-negative breast cancer (TNBC) cells induced by treatment with the hydroalcoholic extract of *T. officinale* showed the inhibition of cell growth and proliferation mediated by the induction of autophagy and apoptosis [11].

Various compounds isolated from *T. officinale* exhibit cytotoxic effects against a range of cancers, including liver, papillary thyroid, promyelocytic leukemia, and cervical carcinoma, with taraxasterols being the primary biological components demonstrated to possess anticarcinogenic properties, particularly against colon and breast cancer by impeding tumor cell invasion and metastasis [10].

Breast cancer, the most common and potentially aggressive cancer among women in Western countries, remains a leading cause of female mortality. Understanding the biological mechanisms underlying cancer progression is crucial for identifying biomarkers and developing targeted treatments. Epigenetic factors play a key role in sustaining tumor advancement, involving processes such as cell detachment, migration, invasion, and metastasis. The diversity of breast cancer forms and their unpredictable evolution are attributed to genetic alterations within the mammary epithelium and influences from the surrounding stromal microenvironment, leading to varied responses to therapies among patients [12].

As reported by Sigstedt et al. [13], the aqueous extract of *T. officinale* root blocks the invasion of MC7/AZ breast cancer cells, while the leaf extract blocks the invasion of LNCaP prostate cancer cells, by reducing the phosphorylation levels of focal adhesion kinase and src, as well as suppressing the activities of matrix metalloproteinases (MMPs), such as the two gelatinases MMP-2 and MMP-9. Metalloproteinases (MMPs) are a group of zinc-dependent endopeptidases essential for extracellular matrix (ECM) reorganization and are involved in numerous human disease [14]. MMPs play an important role in the progression of different tumor types, the process of angiogenesis, and metastatic progression [15,16,17]. The up-regulation of MMPs plays a key role in the release of several molecules involved in the ECM degradation process and consequently in tumor mass invasion [16,18].

Breast cancer involves not only mutated somatic cells but also a complex microenvironment comprising various cell types like fibroblasts, adipocytes, and immune and endothelial cells, which interact closely to regulate cancer pathways. Oxidative stress plays a crucial role in breast cancer initiation and progression, arising from an imbalance between reactive oxygen species and antioxidants, with factors like NOX4 ROS stimulating the migration of cancer cells and myofibroblasts contributing to tumor aggressiveness through collagen secretion and extracellular matrix turnover. Research suggests that detecting elevated ROS levels early could be beneficial in halting cancer progression, exemplified by a study showing increased hydrogen peroxide levels in localized breast cancer patients’ breath condensate correlating with disease severity [19].

Evaluating the effects of natural compounds on the expression and activity of MMPs in 3D cell culture systems can provide valuable insights into their potential to inhibit the metastatic cascade. These more physiologically relevant 3D models can better capture the complex interactions between cancer cells, the ECM, and the tumor microenvironment, which are crucial factors in the metastatic process.

In the present work, for the first time, the phytochemical composition of *T. officinale* aerial parts grown in Umbria, Italy, was carried out by the SPME/GC-MS technique to describe the volatile content, and by GC-MS after the derivatization reaction to characterize the non-volatile fraction. Further, via HRGC-MS chemical investigation, antioxidant activity and the cytotoxic and antiproliferative properties of *T. officinale* methanol along with the dichloromethane extracts of the MDA cells on the breast cancer cell line and on the MCF 10A of normal epithelial cells were evaluated. The effects on the matrix metalloproteinases MMP-2 and MMP-9 were also investigated on the MDA and MCF 10A in 3D cell systems to study the efficacy of these extracts on cell invasiveness.

## 2. Results and Discussion

### 2.1. SPME/GC-MS: Chemical Volatile Composition of Dried T. officinale

By the SPME/GC-MS technique, the volatile fraction was investigated and twenty compounds were identified (Table 1). The chromatogram was also reported (Figure S1). The monoterpene fraction exceeded the sesquiterpene fraction with limonene (31.9%) and 1,8-cineole (38.3%) as the most abundant components. Limonene is known to be a potential natural anticancer compound as it is able to exert chemopreventive activity by limiting tumor growth and angiogenesis in various cancer models [20]. On the other hand, 1,8-cineole has demonstrated potential therapeutic applications through both anti-apoptotic and pro-apoptotic effects [21].

The sesquiterpenes found such as *β*-elemene, *β*-caryophyllene, humulene, and *β*-eudesmene were detected with percentage mean values ranging from 0.4% to 1.3%.

An interesting observation was the presence of dihydroactinidiolide, a lactone compound, which was detected with a relative percentage equal to 7.5%. It was detected in plant leaves and fruits and it was reported to accumulate in *Arabidopsis* leaves under high light stress [22]. It is known to be a potent plant growth inhibitor and is a component of pheromones in insect [23]. Further, this bioactive molecule was also found to exhibit cytotoxic effects against cancer cell lines [24].

Nonanal, an aliphatic aldehyde, was also present in significant quantities (6.1%). It is a component of pheromones in several insect species, playing an essential role in their communication and behavior [25]. Antifungal and antibacterial effects were reported [26,27].

In a previous study conducted by the same technique on seven samples of *T. kok-saghyz* Rodin and four samples of *T. officinale*, several volatile organic compounds (VOCs) and fatty acids were detected with different trends in the investigated roots and leaves. The results of the analyses demonstrated compositional differences between the analyzed samples, and this was mainly linked to the different places of origin of the plants [28].

### 2.2. Chemical Composition of T. officinale Aerial Parts after Derivatization Reaction

The GC-MS analyses conducted on the dried sample after derivatization allowed for the putative identification of fatty acids, triterpenes, and diterpenes (Table 2). The chromatogram was also reported (Figure S2). Among fatty acids, α-linolenic acid (33.1%) and palmitic acid (24.8%) were the main ones followed by linoleic acid (9.1%) and stearic acid (6.9%). This percentage trend is in line with what was reported in the work of Escudero et al., conducted on the leaves of *T. officinale* [29]. This class of compounds was also detected in the seeds of *T. officinale* with linoleic acid being the most abundant, and in the roots where oleic acid prevailed [30,31]. However, fatty acids are found in large quantities mainly in the leaves, indicating the nutritional potential of this *T. officinale* plant tissue [30].

Among the triterpenes detected, lupeol stood out (8.1%) while α-amyrin and *β*-amyrin reached overlapping relative percentage values (1.5 and 1.3%, respectively). In an experimental study conducted on complete plants regenerated from callus cultures of *T. officinale*, amyrins were isolated and found in all tissues, while lupeol was found in differentiated organs [32]. In another study, a protocol was optimized to ensure cell growth and accumulation of *α*-amyrin and lupeol in suspension cultures of *T. officinale* under different culture conditions with the aim of scaling up the production of these triterpenes from *T. officinale* at a large scale [33].

Lupeol is a natural triterpenoid widely present in many medicinal plants. Previous studies conducted on its pharmacological activities have shown that lupeol has antitumoral, antioxidant, anti-inflammatory, and antimicrobial effects [34,35]. *α*-Amyrin and *β*-amyrin are a natural mixture of triterpenes which can be found in numerous plant species [36]. Their potential activities include antitumoral [37], anti-inflammatory [38], and antioxidant effects [39]. Significant relative quantities were found for two diterpene derivatives such as neophytadiene (6.3%) and phytol (4.1%). Finally, another interesting finding is the presence of 2-pyranone (4.8%), an unsaturated cyclic ester, known to be a powerful natural antimicrobial and anticancer agent [40,41].

### 2.3. Chemical Composition of T. officinale MeOH and DCM Extracts

By HRGC-MS analyses carried out on the extracts, eighteen components belonging to different chemical classes were identified (Table 3). The chromatograms were also reported (Figures S3 and S4). The two extracts showed compositional differences from a qualitative point of view. The fatty acid fraction was found to be prevalent in both extracts with palmitic acid and α-linolenic acid being the most abundant. Stearic acid prevailed in the MeOH extract (6.0%) compared with in the DCM extract (3.0%), while linoleic acid prevailed in the DCM extract (14.8%) rather than in the MeOH extract (6.6%). Triterpenes, such as *α*- and *β*-amyrin, and lupeol were present in both extracts with percentage values ranging from 0.6% to 4.7%. On the other hand, triterpene squalene (0.9%) and the diterpenes thunbergol (0.4%) and phytol (0.6%) were only detected in the DCM extract. 2-Pyranone, present only in traces in the DCM extract, reached 22.4% in the MeOH extract. Other qualitative differences were observed in the compositional profile of the two extracts regarding the presence of some alkane compounds that were detected only in the DCM extract and whose relative quantities did not exceed 2.1%.

Previous studies investigated the methanol and the water/ethanol extracts from different parts of *T. officinale* but with the aim to measure the total content of flavonoids and phenols [42,43]. In another work, phytochemical tests were conducted on extracts obtained with various solvents on different parts of *T. officinale* to measure the total content of alkaloids, flavonoids, steroids, saponins, tannins, and triterpenoids. The results showed that the composition varied according to the type of solvent used and the plant tissue investigated [44]. GC/MS technique was used in the work of Sasikala et al. [45], where the ethanolic extract of *T. officinale* leaves were analyzed, showing a composition characterized by aliphatic alkanes and aromatic compounds. Our results are partially in line with what was reported by Razak et al. [46], where a GC/MS analysis of *T. officinale* methanol extract from a mix of leaves, stems, and roots harvested in Kashmir showed the presence of *β*-amyrin and lupeol and fatty acids.

### 2.4. Cytotoxicity Assay

To assess the cytotoxic and antiproliferative activity of the *T. officinale* MeOH and DCM extracts, MDA breast adenocarcinoma cells and MCF 10A non-tumor breast cells were treated in a dose- and time-dependent manner. At all concentrations tested, no significant antiproliferative or cytotoxic effects were observed in all cell lines at 24, 48, or 72 h. MTT assay results indicated constant cell viability at all concentrations and times, suggesting a lack of activity of the extracts under these conditions. The dose–response curve did not show the expected decrease in cell viability with increasing extracts concentrations. EC_50_ values obtained for MDA cells after extract treatments were 867.17 µg/mL ± 8.69 µg/mL and 1075.00 µg/mL ± 17.90 µg/mL for MeOH and DCM, respectively. The lack of antiproliferative and cytotoxic effects of the *T. officinale* MeOH and DCM extracts in the MTT assay suggests that the extracts had no cytotoxic properties against the cells considered, having obtained EC_50_ values > 250 µg/mL. In contrast, the cytotoxic effects of *T. officinale* have been documented across various tumor cell lines, as reported in a comprehensive review detailing the antitumor properties of extracts derived from the parts of this plant and by various extraction solvents, highlighting antitumor activities linked to apoptotic mechanisms and cell cycle regulation [11,47].

In MCF-7 breast cancer cell lines, the EC_50_ value of 190.5 µg/mL was recognized after treatment with ethanolic plant extracts [48]. In other cell lines such as HL60, *T. officinale* stem extracts were found to be more active than leaf extracts, with EC_50_ ranging from 71 µg/mL to 540 µg/mL, respectively. However, as reported by Indrayanto and colleagues, EC_50_ values > 100 µg/mL indicate that a plant extract is inactive [47].

Similarly, Trinh et al. [49] tested ethanol and methanol dandelion extracts on breast cancer stem cells (BCSCs) and found the inhibition of proliferation in both 2D and 3D models with the IC_50_ value in the 3D model much higher than that in the 2D model [49].

### 2.5. Antioxidant Activity

**Figure 1:**
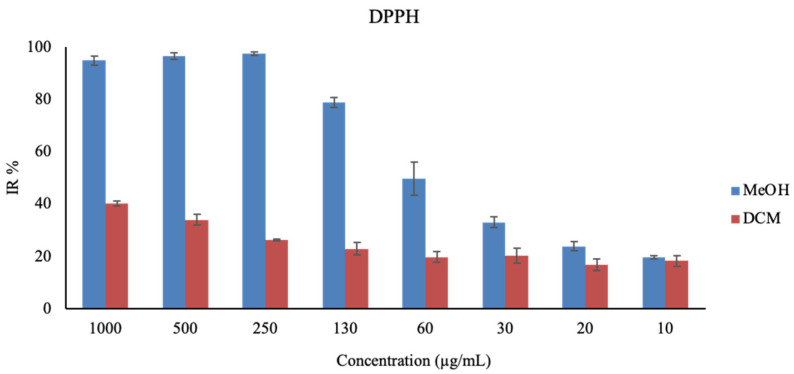
Graph bar of the antioxidant capacity of MeOH and DCM extract results obtained by the DPPH assay. Percentages of scavenged free radicals correlated with different concentrations of extracts, expressed in μg/mL.

DCM and MeOH extracts were tested to measure their antioxidant activity. The antioxidant capacity was assessed using two colorimetric assays, and the findings are detailed in Table 4. The MeOH extract exhibited notable antioxidant activity with an IC_50_ value of 0.04 ± 0.00 mg/mL, or 0.35 ± 0.04 Trolox equivalent (TEAC), while the DCM extract showed an IC_50_ of 2.47 ± 0.19 mg/mL, or 0.08 ± 0.00 TEAC, as determined by the DPPH assay. The graph bar of the % of scavenged free radicals at different concentrations obtained by DPPH is reported in Figure 1. In the ferric-reducing antioxidant activity (FRAP) assay, the MeOH extract demonstrated an antioxidant activity of 220.65 ± 8.68 μmol Trolox equivalent per gram of dry weight (μmol TE/g DW). Similarly, the DCM extract displayed an antioxidant activity of 208.06 ± 1.79 μmol TE/g DW (Table 4).

The DPPH assay showed greater antioxidant activity for the MeOH extract than for the DCM extract, while the FRAP assay showed the opposite, with the DCM extract having greater, albeit slight, antioxidant capacity. The contrasting results from the DPPH and FRAP assays, indicating different antioxidant capacities for the extracts, suggest that *T. officinale* likely contains a range of antioxidants with distinct mechanisms of action. This aligns with other studies that have highlighted the complex antioxidant profile of this plant, attributing its efficacy to a variety of bioactive compounds, including polyphenols and polysaccharides [50,51]. The IC_50_ and TEAC values detected by DPPH demonstrated a higher antioxidant activity of the MeOH and DCM extracts compared with the ethanolic and hydroethanolic leaf extracts, which presented 0.21 mg/mL and 0.40 mg/mL, respectively [52,53].

### 2.6. MMP-9 and MMP-2 Detection by Gelatin Zymography and Western Blot Analyses

Among the various metalloproteinases that have been identified, the study focused on MMP-9 and MMP-2 because they are known to be consistently expressed in many different types of connective tissue cells. These metalloproteinases, also referred to as gelatinases, have the ability to remodel the extracellular matrix and break down a wide range of collagen types. They are actively involved in the processes of tumor mass invasion [11,54].

**Figure 2:**
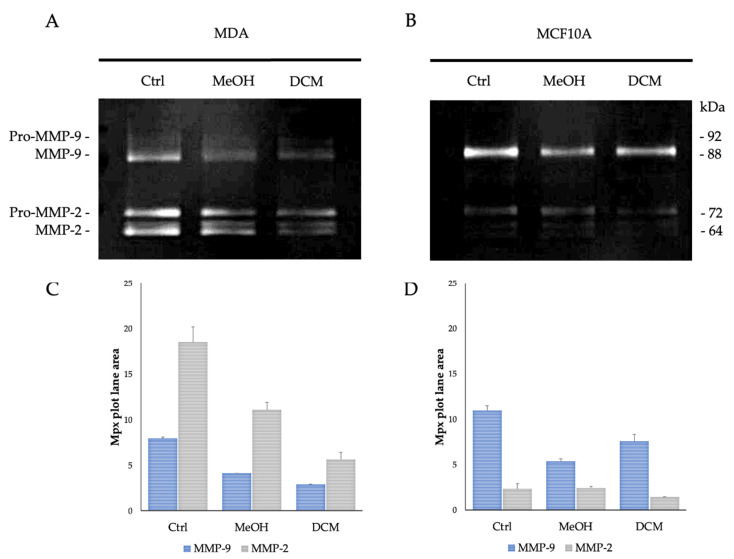
Gelatin zymography on MDA and MCF10A: (**A**) MDA and (**B**) MCF 10A zymograms performed after treatment with MeOH and DCM extracts. The cells cultured in a 3D collagen matrix revealed the presence of two major protein bands. These are identifiable based on their respective molecular weights as pro-MMP-9 and active MMP-9 (92 and 88 kDa, respectively) and pro-MMP-2 and active MMP-2 (72 and 64 kDa, respectively). (**C**) MDA and (**D**) MCF 10A zymograms scanned with a densitometer and quantification of the different signal intensities detected.

To assess any modulatory effects of the extracts on the regulation of the two gelatinases expressed by MDA and MCF 10A cells cultured in 3D systems, a zymography was performed on SDS enriched with gelatin (Figure 2).

In the 3D collagen matrix, both cell lines demonstrated distinct expression patterns of MMP-9 and MMP-2, with higher MMP-9 expression in the non-tumor cells and elevated MMP-2 expression in the tumor cells.

In the MDA adenocarcinoma cell line, MMP-9 levels appeared to be approximately halved after 48 h of treatment with MeOH and DCM extracts at 4.11 ± 0.00 Mpx and 2.92 ± 0.03 Mpx, respectively, compared with untreated cells (Ctrl) as revealed in the zymography quantification (7.94 ± 0.17 Mpx). The MMP-2 levels were reduced from 18.48 ± 1.69 Mpx in the Ctrl sample to 11.08 ± 0.86 Mpx after MeOH treatment and to 5.64 ± 0.80 Mpx after DCM extract treatment (Figure 2A,C).

In the MCF 10A non-tumor cell line, the MeOH extracts reduced MMP-9 levels from 11.00 ± 0.53 Mpx to 5.37 ± 0.27 Mpx, whereas the DCM-treated sample showed a partial reduction in the corresponding gelatinase level (from 7.58 ± 0.80 Mpx). MMP-2 appeared poorly expressed in this cell line, for all three samples considered, treated (MeOH and DCM extracts) and untreated (Ctrl), ranging from 2.35 ± 0.60 Mpx to 1.45 ± 0.04 Mpx (Figure 2B,D).

**Figure 3:**
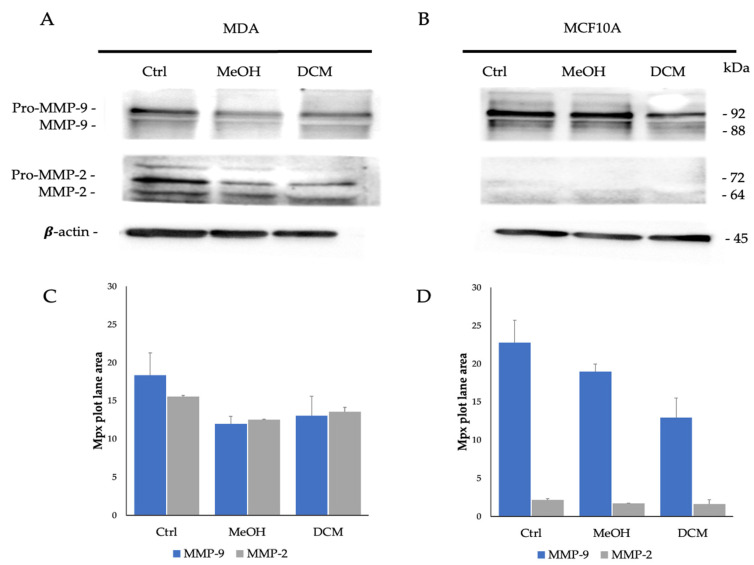
Western blot analysis on MDA and MCF10A treated with MeOH and DCM extracts. (**A**,**B**) Immune detection of MMP-9 (92 and 88 kDa), MMP-2 (72 and 64 kDa), and b-actin (45 kDa). (**C**,**D**) Bar graphs of the intensity of the detected bands.

Therefore, the results of the gelatin zymography assay conducted on MDA and MCF 10A cells cultured in 3D systems and treated with MeOH and DCM extracts revealed significant insights into the modulation of gelatinase expression levels, with a higher activity on MMP-2 for DCM with respect to MeOH extract. Following a 48 h treatment period, significant alterations in gelatinase levels were observed. For the MDA adenocarcinoma cell line, MMP-9 levels were notably reduced by approximately half post-treatment with MeOH and DCM extracts compared with control cells, as evidenced by zymography quantification. Similarly, MMP-2 levels exhibited reductions after treatment with both extracts compared with the control sample. In contrast, the MCF10-A non-tumor cell line displayed reduced MMP-9 levels after treatment with MeOH extracts, with a partial reduction observed in DCM-treated samples. MMP-2 expression remained consistently low across all samples for this cell line. These findings underscore the potential modulatory effects of the MeOH and DCM extracts on the regulation of gelatinases in the tested cell lines, suggesting a differential impact on MMP-9 and MMP-2 expression levels in tumor and non-tumor cell contexts in a 3D culture environment. When detected by anti-MMP-9 and anti-MMP-2 antibodies in Western blot analysis (Figure 3), tumor and non-tumor cell lines cultured in a 3D collagen matrix showed different expressions of the two metalloproteinases.

The treatment of the MDA cell line with MeOH and DCM extracts showed a reduction in the bands detected for both metalloproteinases with respect to the untreated cells. By quantification analysis (Figure 3C), the 11.94 ± 1.01 Mpx and 13.05 ± 2.57 Mpx intensity values for MMP-9 and 12.52 ± 0.05 Mpx and 13.57 ± 0.56 Mpx for MMP-2 were observed for treatment with the MeOH and DCM extracts, respectively, while in Ctrl cells, they were 18.33 ± 2.93 Mpx for MMP-9 and 15.55 ± 0.56 Mpx for MMP-2.

As obtained by gelatin zymography analysis, the expression of MMP-9 was higher in MCF 10A than in MDA. In the normal lines, treatment with MeOH extract resulted in a slight decrease in the intensity of expression, ranging from 22.77 ± 0.87 Mpx (untreated cells) to 18.94 ± 2.27 Mpx. In contrast, an intensity of 12.94 ± 0.38 Mpx was observed in DCM extract treatment. A low intensity of MMP-2 was revealed on treated and untreated normal cells.

The Western blot analysis using anti-MMP-9 and anti-MMP-2 antibodies unveiled distinct expressions of these metalloproteinases. In the MDA cell line, treatment with MeOH and DCM extracts led to reductions in the bands detected for both MMP-9 and MMP-2 compared with untreated cells, as shown by the quantification analysis. Contrary to MDA cells, MCF 10A cells exhibited higher MMP-9 expression levels. Treatment with the MeOH extract in the non-tumor cells resulted in a slight decrease in MMP-9 expression intensity, while DCM extract treatment showed a more significant reduction. MMP-2 expression remained low in both treated and untreated normal cells. These results indicate that the MeOH and DCM extracts have differential effects on MMP-9 and MMP-2 expression levels in tumor and non-tumor cell lines within a 3D culture setting. Similar activity was revealed for both extracts. Therefore, the evidence obtained by gelatin zymography and Western blot analysis showed a modulatory effect on MMP-9 and MMP-2 activity and expression.

As reported by Mukherjee et al. [55], several plant extracts such as *Salvia miltiorrhiza*, *Paris polyphylla* Sm., *Elaeagnus glabra* K.Koch, *Pothomorphe umbellate* (L.) Miq., *Magnolia officinalis* Rehder and E.H.Wilson, *Salicornia herbacea* (L.) L., *Artemisia capillaris* Miq., *Citrus depressa* Hayata, *Selaginella tamariscina* (P.Beauv.) Spring, and *Metasequoia glyptostroboides* Hu and W.C.Cheng have been found to inhibit the activity of MMP-9 and MMP-2, thus impacting cancer cell invasiveness, metastasis, and angiogenesis through various molecular mechanisms [55]. Moreover, different classes of natural compounds from terrestrial and marine sources such as long-chain phenolic lipids, polysaccharides, flavonoids, and polyphenols, as well as small molecules and terpenoids, have been reported to inhibit MMPs or reduce MMP expression, which are involved in different pathological condition [56].

A regulatory role on the modulation of MMP-9 and MMP-2 levels was observed in breast cancer cell lines (MCF-7/AZ) following treatment with aqueous extracts from leaves of different parts of *T. officinale* as opposed to LNCaP C4-2 B prostate cancer cells that remained unaffected [6].

In breast cancer, a significant proportion of stromal cells undergo a pivotal activation process marked by the increased release of growth factors, cytokines, and metalloproteinases. Furthermore, these cells actively produce hydrogen peroxide, setting off a cascade that not only engenders more activated cells but also instigates tumorigenic alterations in adjacent cells, further propelling the progression of the disease [55,57]. The process of both collagen formation and breakdown is crucial for the natural restructuring of connective tissue during cellular growth, maturation, and the infiltration of cancerous cells. Enzymes like MMP-2, MMP-3, and MMP-9 enhance the turnover of the extracellular matrix and are activated in response to oxidative stress [17].

Nicolai and colleagues [58] have shown that the Omega-3 (including α-linolenic acid) and Omega-6 (including linoleic acid) fatty acids have an inhibitory proteolytic activity of MMP-2 and MMP-9 that participate in the destruction of the organic matrix of dentin, following demineralization operated by bacterial acids.

The plant-derived compounds could offer valuable tools for averting the invasion and spread of breast cancer. The utilization of natural agents that inhibit MMPs is being explored as a way to innovate alternative treatment approaches for breast cancer, enhancing traditional treatments by targeting multiple aspects simultaneously [54].

## 3. Materials and Methods

### 3.1. Plant Material

*Taraxacum officinale* Weber (ex F.H. Wigg. 1780) dried aerial parts (Lot reference No. 119/22/230123/A) were purchased from the company “Erbamea” (Umbria, Italy).

### 3.2. SPME Sampling

To describe the volatile chemical profile of the dried aerial parts of *T. officinale*, SPME sampling technique was used. About 0.5 g of the sample was placed inside a 7 mL glass vial with a PTFE-coated silicone septum. To collect the volatiles from the headspace of the matrix, an SPME device from Supelco (Bellefonte, PA, USA) with 1 cm fiber coated with 50/30 μm DVB/CAR/PDMS (divinylbenzene/carboxen/polydimethylsiloxane) was used [59]. Before use, the fiber was conditioned at 270 °C for 30 min. To capture and pre-concentrate the volatiles, the fiber sampled for 30 min at 50 °C. Lastly, for the desorption phase, the SPME fiber was inserted into the GC injector and maintained at 250 °C in splitless mode.

### 3.3. GC-MS Analysis

To carry out the chromatographic analyses, a Clarus 500 model Perkin Elmer (Waltham, MA, USA) gas chromatograph equipped with an FID (flame ionization detector) and coupled with a mass spectrometer was used. The chosen capillary column was a Varian Factor Four VF-5. The GC oven’s programmed temperature was set initially at 45 °C for 2.0 min and then increased to 220 °C at 6°/min and held for 15 min. Helium was used as carrier gas at a constant rate of 1 mL/min. MS detection was performed with electron ionization (EI) at 70 eV operating in the full-scan acquisition mode in the *m*/*z* range of 35–500 amu. The identification of compounds was performed by the comparison of the MS-fragmentation pattern of the analytes with those of pure components stored in the Wiley 2.2 and Nist 11 mass spectra libraries’ databases. Further, the Linear Retention Indices (LRIs) were calculated using a series of alkane standards (C_8_–C_40_ *n*-alkanes). The obtained LRIs were compared with those available reported in the literature. For the quantification, the relative amounts of the components were expressed as percent peak area relative to total peak area without the use of an internal standard and any factor correction. The analysis was carried out in triplicate.

### 3.4. GC-MS Analysis of Taraxacum officinale after the Derivatization Reaction

To describe the chemical composition of the *T. officinale* dried aerial parts, a derivatization reaction was performed according to Taiti et al. [60], with slight modifications. The analysis was performed using the same apparatus GC-FID/GC-MS and the same capillary column (Varian Factor Four VF-5). Approximately 0.5 mg of matrix was added to 300 μL of pyridine and 100 μL of bis-(trimethylsilyl) trifluoroacetamide (BSTFA) with heating at 80 °C for 120 min. A total volume of 1 μL of the silylated sample was manually injected at 280 °C into the GC injector in splitless mode. The applied program temperature was as follows: 80 °C, then a gradient of 7 °C/min up to 170 °C held for 1.0 min and a gradient of 8 °C/min up to 250 °C held constant for 25 min. Mass spectra were acquired in electron impact mode. The identification of the compounds was performed considering the percentage of similarity with the mass spectra (MS) present in the instrument library database (NIST 11). Quantification was performed as described above (Section 3.3).

### 3.5. Extraction Process

A total of 9.5 Grams of plant material was successively extracted by Soxhlet apparatus with solvents of increased polarity, i.e., dichloromethane (DCM) and methanol (MeOH), in 8 h each. The solvents were evaporated at reduced pressure via Rotavapor at a temperature of 35 °C (for DCM) and 40 °C (for MeOH), obtaining two extracts with a yield of 3.77 and 7.60%.

### 3.6. HRGC-MS Analysis of MeOH and DCM Extracts

To detect the content of fatty acids, diterpenes, and triterpenes, the extracts were analyzed using high-resolution gas chromatography (HRGC/MS). A total volume of 1 μL of each extract was manually injected at 320 °C into the GC injector in the split mode (1:20) [61]. For the detection and good separation of high-molecular-weight molecules, the column oven temperature was programmed from 100 °C to 170 °C at the rate of 7 °C/min, then a gradient of 8 °C/min up to 270 °C held for 1.0 min and a gradient of 10 °C/min up to 300 °C held constant for 20 min. Identification and quantification were performed as reported in the previous section. The analyses were carried out in triplicate.

### 3.7. Cell Lines and Culture Conditions

Human adenocarcinoma breast cell line (MDA, ATCC MB-231, American Type Culture Collection, Manassas, VA, USA) and normal breast epithelial cell line (MCF10A, ATCC CRL-10317) were used to determine the cytotoxic activity of the *T. officinale* MeOH and DCM extracts.

MDA cells were cultured in DMEM F-12 supplemented with 10% fetal bovine serum, 1% glutamine, and 1% penicillin–streptomycin.

MCF10A were maintained in DMEM F-12 medium supplemented with 100 ng/mL cholera toxin, 20 ng/mL epidermal growth factor (EGF), 0.01 mg/mL insulin, 500 ng/mL hydrocortisone, 5% horse serum (HS), 1% glutamine, and 1% penicillin–streptomycin. Before being seeded for the viability assay, the culture medium was deprived of EGF and the HS was reduced to 2%. All the cell lines were cultured at 37 °C in a humidified 5% CO_2_ condition. All experiments were performed with cells in their logarithmic growth phase.

#### Preparation of 3D RAFT™ Cultures

Once the growth phase was reached, the MDA and MCF10A cells were used for 3D cell cultures in the RAFT™ system. The cells were seeded in a 24-well plate of the RAFT™ system according to the manufacturer’s instructions (TAP Biosystems, Lonza, Cologne, Germany). Briefly, cells were dispensed in the chilled mixed collagen solution of the RAFT™ kit (containing 10X MEM medium, 2 mg/mL rat tail collagen type I, neutralizing solution) according to the RAFT™ protocol. A total volume of 2 mL of mixed collagen solution was placed in each well and incubated for 15 min at 37 °C to form a hydrogel. Then, the RAFT™ absorber was placed on top of the hydrogel in a laminar flow hood at room temperature (RT) for 15 min. After removing the absorbers, immediately, 2 mL of the respective culture medium was loaded in each well. The medium was replaced every three days. After 14 days of incubation, the 3D cells were treated with 0.5 mg/mL of *T. officinale* MeOH extracts and DCM, while the 3D cells without treatment were used as controls.

### 3.8. Cytotoxicity and Antiproliferative Assay

To define the cytotoxic and antiproliferative activity of the *T. officinale* MeOH and DCM extracts, the MTT assay was carried out on 2D cell culture as described by Letaief et al. [62]. To perform the assay, 10,000 cells/well of every cell line were seeded in a 96-well plate. After 24 h, cells were treated with 100 uL of the culture medium containing decreasing concentrations of each extract, starting from 1 mg/mL and followed by 10 serial dilutions of 1:2 for a final volume of 200 μL/well. DMSO was used as solvent control.

At the 24 and 48 h time points, the culture medium containing the treatment was removed and 100 μL of a 0.5% MTT solution in the culture medium was added and incubated in the dark at 37 °C for 3 h. After the incubation time, dimethyl sulfoxide (DMSO) was added to solubilize the formed formazan crystals. The plates were incubated at 37 °C in the dark for an additional 30 min. Finally, the plates were read at 570 nm using a Sunrise Tecan microplate reader. This measurement allows for the quantification of the formazan crystals formed by the reduction in MTT by viable cells, indicating cell viability or metabolic activity:

$$Cell\;viability\%=\frac{Average\;absorbance\;of\;treated\;cells}{Average\;absorbance\;of\;untreated\;cells\;(Ctrl)}\times 100$$

The results were analyzed using GraphPad Prism version 8.0.2 for Windows (GraphPad Software, La Jolla, CA, USA). The half-maximal concentration (EC_50_) for each biological replicate was determined using a constructed log–response curve. Overall, EC_50_ was calculated from three independent biological replicates.

### 3.9. Antioxidant Assays

To define the antioxidant activities of the *T. officinale* MeOH and DCM extracts, DPPH and FRAP assays were performed.

#### 3.9.1. DPPH Assay

The microplate method for the removal of DPPH radicals was used to test the antioxidant efficacy of the extracts. In a 96-well microplate, 20 μL of sample diluted at different concentrations were combined with 180 μL of 0.15 mM DPPH solution. Subsequently, absorbance was measured at 515 nm after a 40 min incubation period in the absence of light at room temperature using a Tecan SunriseTM UV-vis spectrophotometer. Results, conducted in triplicate, were reported as the percentage of inhibition of DPPH absorbance in relation to the control values without extract. A reduction in the absorbance of the DPPH solution signifies a rise in the scavenging prowess of the DPPH radical, quantified as the percentage of inhibition concentration (IC%) through the following formula:

IC% = Ac − As/Ac × 100

where Ac represents the absorbance of the control and As stands for the absorbance of the sample. By gauging the percentage scavenging capabilities across various concentrations, the antioxidant effectiveness of the samples was assessed by determining the effective concentration or the quantity (mg/mL) of the samples necessary to neutralize 50% (IC_50_) of the radical. IC_50_ values of the samples and Trolox (mM) were calculated using calibration curves. The values, expressed in Trolox equivalent (TEAC), were obtained from the ratio between Trolox IC_50_ and sample IC_50_.

#### 3.9.2. FRAP Assay

The FRAP assay is based on the ability of a substance to reduce Fe^3+^ to Fe^2+^. The levels of Fe^2+^ produced were evaluated by spectrophotometrically measuring, at 595 nm, the colored complex it forms with 2,4,6-Tris(2-pyridyl)-s-triazine (TPTZ).

In 96-well microplates, 20 μL of the appropriate concentration of samples and Trolox dilutions were mixed with 180 μL of the FRAP solution, according to Xiao et al. [63]. Following a 40 min incubation at 37 °C, absorbance at 595 nm was measured using a Tecan SunriseTM UV-vis spectrophotometer. Trolox solutions, at concentrations ranging from 2 to 0.002 mM, were used to construct the standard curve.

Triplicate assays were performed and the results were expressed as μmol TE/g DW as described in the previous assay.

### 3.10. Gelatin Zymography

Since zymography measures enzymatic activity after denaturation and renaturation of the enzymes, it can measure the activity of all MMPs present in the sample. The presence of SDS exposes the active site of the enzyme that can initiate the degradation of gelatin after partial renaturation. MMP-9 is secreted as a 92 kDa precursor and activated upon release of 82 kDa, while MMP-2 is secreted as a 72 kDa precursor and activated upon release of 64 kDa.

To detect the MMPs’ activity in 2D and 3D culture samples, the protocol of Toth and Fridman [64], for gelatin zymography, was routinely used. To maintain enzymatic activity, the samples must be electrophoresed under non-reducing conditions and without boiling.

Samples were electrophoresed in a 10% SDS-polyacrylamide gel containing 0.1 mg/mL of porcine gelatin (Sigma-Aldrich, St. Louis, MI, USA) and subsequently incubated for 30 min at room temperature in a 2.5% (*v*/*v*) Triton X-100 solution for enzyme renaturation. Following a thorough rinse in double distilled water, gels were incubated at 37 °C for 20 h in developing buffer (50 mM Tris-HCL buffer, pH 7.8 containing 20 mM sodium chloride, 5 mM calcium chloride, and 0.02% Brij-35), stained with 0.1% Coomassie Brilliant Blue R-250, and destained in 10% acetic acid and 30% methanol.

Gelatinolytic activity was detected as unstained bands on a blue background and revealed with the ChemiDoc instrument (Bio-Rad, Hercules, CA, USA). Acquired pictures were quantitatively evaluated using the ImageJ program version ImageJ 1.48v—Wayne Rasband, National Institute of Health, Bethesda, MD, USA.

### 3.11. MMP-9 and MMP-2 Detection by Western Blot

The 3D MDA cell culture samples were treated for 24 h with 0.5 mg/mL of *T. officinale* MeOH and DCM extracts. All samples were collected in a lysis buffer NP-40 buffer (20 mM Tris–HCl, 165 mM NaCl, 5 mM EDTA and 1% *v*/*v* NP-40, pH 7.5) with protease inhibitors added. The obtained protein concentration was determined using the Bradford micro-assay method (Sigma-Aldrich).

A total of 20 µg/mL of protein for each sample was loaded on a 10% sodium dodecyl sulfate (SDS)-polyacrylamide gel. To detect antibody-binding proteins, samples were transferred into nitrocellulose membranes (Millipore, Burlington, MA, USA) using a semi-dry transfer system (Bio-Rad, Hercules, CA, USA) and transfer buffers. These membranes were then blocked in Tris-buffer saline with 0.01% Tween-20, pH 7.4 with 5% BSA (TBST) for 1 h at room temperature, washed, and incubated overnight at 4 °C with a rabbit polyclonal antiserum anti-MMP-9 (Ab 38898, Abcam, Cambridge, MA, USA) and a rabbit polyclonal antiserum anti-MMP-2 (Ab 37150, Abcam) diluted at 1:1000. Nitrocellulose membranes were washed and incubated for 4 h with a horseradish peroxidase-conjugated goat anti-rabbit IgG secondary antibody diluted at 1:1000 with TBST and then developed using the ECL chemiluminescence detection system (ChemiDoc, Bio-Rad). Acquired pictures were quantitatively evaluated using the ImageJ program version ImageJ 1.48v—Wayne Rasband, National Institute of Health, USA.

### 3.12. Statistical Analysis

The mean and standard deviation (SD) of replicates were calculated. Statistical analysis was preformed using a one-way ANOVA test with a Stat-Plus software (AnalystSoft v8) with the threshold of significance set at *p* < 0.05.

## 4. Conclusions

This study highlighted how the aerial parts of *Taraxacum officinale* F.H. Wigg are sources of numerous secondary metabolites with high bioactive potential belonging to different chemical classes such as, mono- e sesquiterpenes, di- and triterpenes, and fatty acids. The MeOH and DCM extracts resulted to possess the ability to influence the expression of key metalloproteinases in a cell-type-specific manner within a 3D culture environment. These effects are certainly linked to the pool of metabolites detected in the extracts, which perform their bioactive potential. The modulation of MMPs represents an encouraging field of study that may lead to the creation of novel therapeutic possibilities for different types of breast cancer.

The analysis of metalloproteinase expression levels in tumor (MDA) and non-tumor (MCF 10A) cell lines cultured in a 3D collagen matrix following treatment with MeOH and DCM extracts represent a novelty into the modulatory effects of these extracts on MMP-9 and MMP-2. However, further research into the mechanisms of action and their implications for possible therapeutic applications could provide valuable insights into the utility of these extracts in modulating metalloproteinase activity in cancer cells.

## Figures and Tables

**Table 1:** Chemical volatile composition (percentage mean values ± standard deviation) of *T. officinale* dried aerial parts, as determined by SPME-GC/MS.

N°	Component ^1^	LRI ^2^	LRI ^3^	Content (%)
1	α-pinene	928	932	2.1 ± 0.03
2	β-pinene	978	981	0.8 ± 0.02
3	β-myrcene	982	987	1.8 ± 0.02
4	limonene	1020	1022	31.9 ± 0.15
5	1,8-cineole	1031	1033	38.3 ± 0.20
6	linalool	1087	1089	1.2 ± 0.02
7	nonanal	1100	1104	6.1 ± 0.04
8	isothujol	1162	1165	1.6 ± 0.03
9	terpinen-4-ol	1178	1182	0.6 ± 0.02
10	1-octanol, 2-butyl-	1270	1277	1.0 ± 0.01
11	isobornyl acetate	1281	1286	0.2 ± 0.02
12	β-elemene	1395	1393	1.3 ± 0.02
13	tetradecane	1410	1413	1.5 ± 0.03
14	β-caryophyllene	1430	1435	1.0 ± 0.01
15	humulene	1468	1473	0.5 ± 0.02
16	β-eudesmene	1479	1485	0.4 ± 0.02
17	dihydroactinidiolide	1520	1525	7.5 ± 0.04
18	hexadecane	1609	1612	0.9 ± 0.03
19	2,6-diisopropylnaphthalene	1725	1728	0.7 ± 0.03
20	hexahydrofarnesyl acetone	1840	1846	0.6 ± 0.02
	monoterpenes			78.5
	sesquiterpenes			3.2
	others			18.3
	SUM			100.0

^1^ The components are reported according to their elution order on apolar column; ^2^ Linear Retention Indices measured on the apolar column; ^3^ Linear Retention Indices from the literature (Nist WebBook); tr: percentage mean values < 0.1%.

**Table 2:** Chemical composition (percentage mean values ± standard deviation) of *T. officinale* dried aerial parts after derivatization, as determined by GC–MS.

N°	Components	(%)
***Fatty acids***		
1	palmitic acid	24.8 ± 0.15
2	oleic acid	tr
3	linoleic acid	9.1 ± 0.08
4	*α*-linolenic acid	33.1 ± 0.22
5	stearic acid	6.9 ± 0.07
***Triterpenes***		
6	lupeol	8.1 ± 0.10
7	*α*-amyrin	1.5 ± 0.02
8	*β*-amyrin	1.3 ± 0.04
***Diterpenes***		
9	neophytadiene	6.3 ± 0.08
10	phytol	4.1 ± 0.03
***Others***		
11	2-pyranone	4.8 ± 0.05

tr: Percentage means values < 0.1%.

**Table 3:** Chemical volatile composition (percentage mean values ± standard deviation) of *T. officinale* extracts.

N°	Component ^1^	LRI ^2^	LRI ^3^	Content (%)	Content (%)
				MeOH Extract	DCM Extract
1	2-pyranone	1110	1107	22.4 ± 0.12	tr
2	tetradecane, 2,6,10-trimethyl-	1552	1557	-	0.5 ± 0.01
3	hexadecane	1609	1612	-	0.5 ± 0.00
4	hydroxylauric acid	1810	1813	0.9 ± 0.03	-
5	neophytadiene	1832	1836	4.9 ± 0.05	8.7 ± 0.05
6	palmitic acid	1958	1962	28.9 ± 2.15	33.2 ± 4.30
7	thunbergol	2028	2032	-	0.4 ± 0.01
8	phytol	2101	2105	-	0.6 ± 0.03
9	3,7,11,15-tetramethyl-2-hexadecen-1-ol	2112	2116	-	2.1 ± 0.02
10	oleic acid	2145	2147	-	0.3 ± 0.01
11	linoleic acid	2148	2152	6.6 ± 0.07	14.8 ± 0.03
12	*α*-linolenic acid	2162	2159	28.2 ± 0.11	26.5 ± 0.08
13	stearic acid	2170	2166	6.0 ± 0.06	3.0 ± 0.03
14	erucic acid	2568	2572	-	0.2 ± 0.01
15	squalene	2541	2847	-	0.9 ± 0.02
16	lupeol	3272	3270	0.6 ± 0.02	4.7 ± 0.05
17	*β*-amyrin	3342	3337	0.8 ± 0.03	1.0 ± 0.02
18	*α*-amyrin	3381	3376	0.6 ± 0.02	0.8 ± 0.02
	fatty acids			70.6	78.0
	triterpenes			2.0	7.4
	diterpenes			4.9	9.7
	others			-	3.1
	SUM			99.9	98.2

^1^ The components are reported according to their elution order on the apolar column; ^2^ Linear Retention Indices measured on the apolar column; ^3^ Linear Retention Indices from the literature (Nist WebBook); tr: percentage means values < 0.1%.

**Table 4:** Antioxidant capacity of MeOH and DCM extracts. DPPH IC_50_ values are expressed in μg/mL and in TEAC (µmol/g DW); FRAP values are expressed in µmol TE/g DW.

	DPPH	FRAP
MeOH	0.04 × 10^3^ ± 0.01 × 10^3^	220.65 ± 8.68
DCM	2.47 × 10^3^ ± 0.19 × 10^3^	208.06 ± 1.79

Data are expressed as the mean ± standard deviation. *p* < 0.05.

## Data Availability

Data will be available on request.

## Supplementary Materials

The following supporting information can be downloaded at: https://www.mdpi.com/article/10.3390/plants13192829/s1, Figure S1: SPME-GC/MS Chromatogram of *T. officinale* dried aerial parts; Figure S2: GC/MS Chromatogram of *T. officinale* dried aerial parts after derivatization; Figure S3: GC/MS Chromatogram of *T. officinale* DCM extract; Figure S4: GC/MS Chromatogram of *T. officinale* MeOH extract.
