# Supplementary material for: Exploring the Bioactive Potential of Taraxacum officinale F.H. Wigg Aerial Parts on MDA Breast Cancer Cells: Insights into Phytochemical Composition, Antioxidant Efficacy, and Gelatinase Inhibition within 3D Cellular Models
Source: Plants (Basel). 2024 Oct 9;13(19):2829. doi: 10.3390/plants13192829 (PMC11482471; doi:10.3390/plants13192829)
Supplement: Supplementary file 1 [file plants-13-02829-s001.zip › plants-3195517-supplementary.pdf]

plants

MDPI

Article

Exploring the Bioactive Potential of *Taraxacum officinale* F.H. Wigg Aerial Parts on MDA Breast Cancer Cells: Insights into Phytochemical Composition, Antioxidant Efficacy, and Gelatinase Inhibition within 3D Cellular Models

Valentina Laghezza Masci [1], Elisa Ovidi [1], William Tomassi [1], Daniela De Vita [2] and Stefania Garzoli [3,*]

[1] Department for Innovation in Biological, Agro-Food and Forest Systems (DIBAF), University of Tuscia, Largo dell'Università, 01100 Viterbo, Italy; laghezzamasci@unitus.it (V.L.M.); eovidi@unitus.it (E.O.); william.tomassi@outlook.com (W.T.)

[2] Dipartimento di Biologia Ambientale, Università di Roma "La Sapienza", Piazzale Aldo Moro 5, 00185 Rome, Italy; daniela.devita@uniroma1.it

[3] Department of Chemistry and Technologies of Drug, Sapienza University, Piazzale Aldo Moro 5, 00185 Rome, Italy

* Correspondence: stefania.garzoli@uniroma1.it

Supplementary Materials

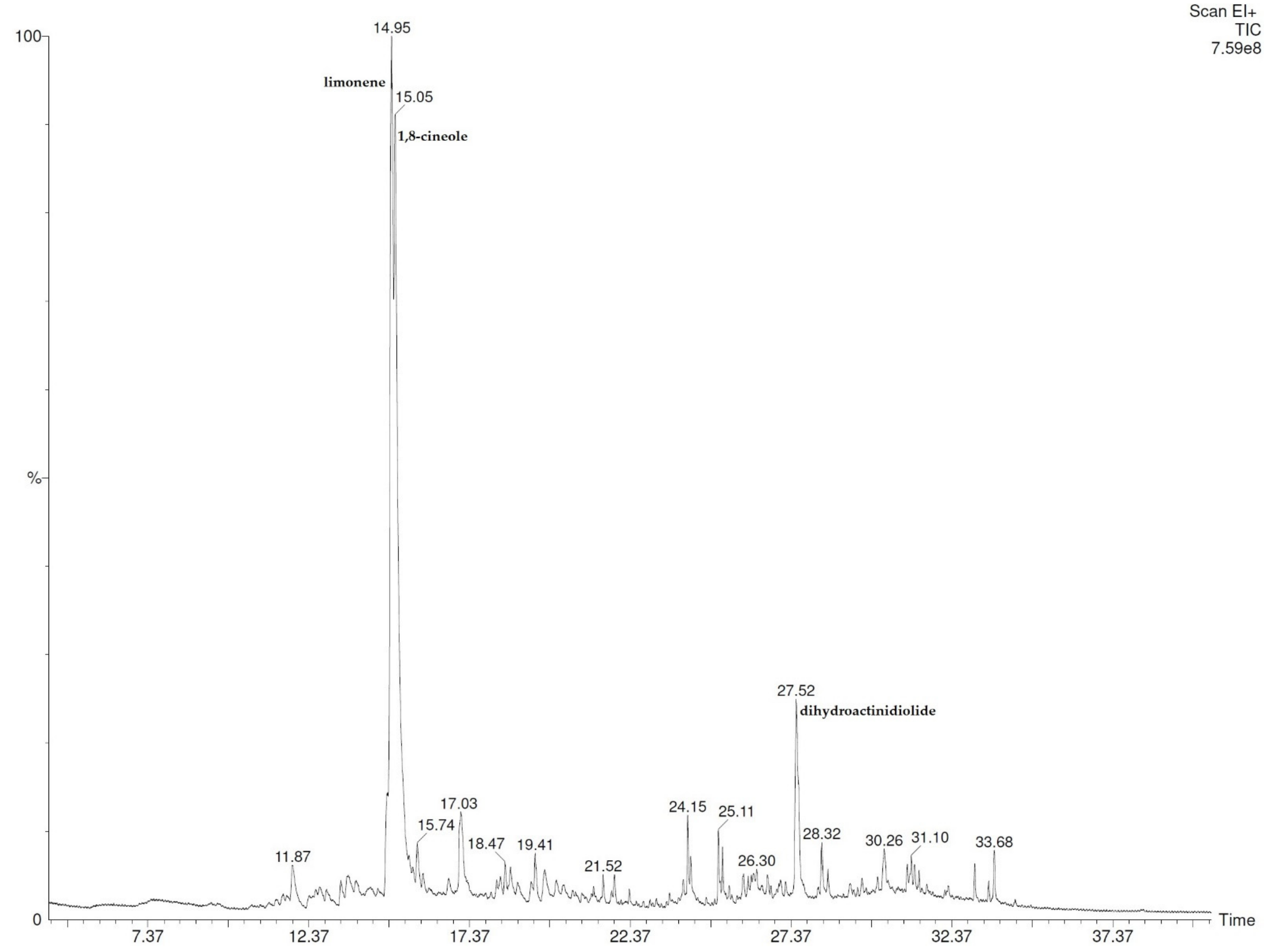

Figure S1. SPME-GC/MS Chromatogram of *T. officinale* dried aerial parts.

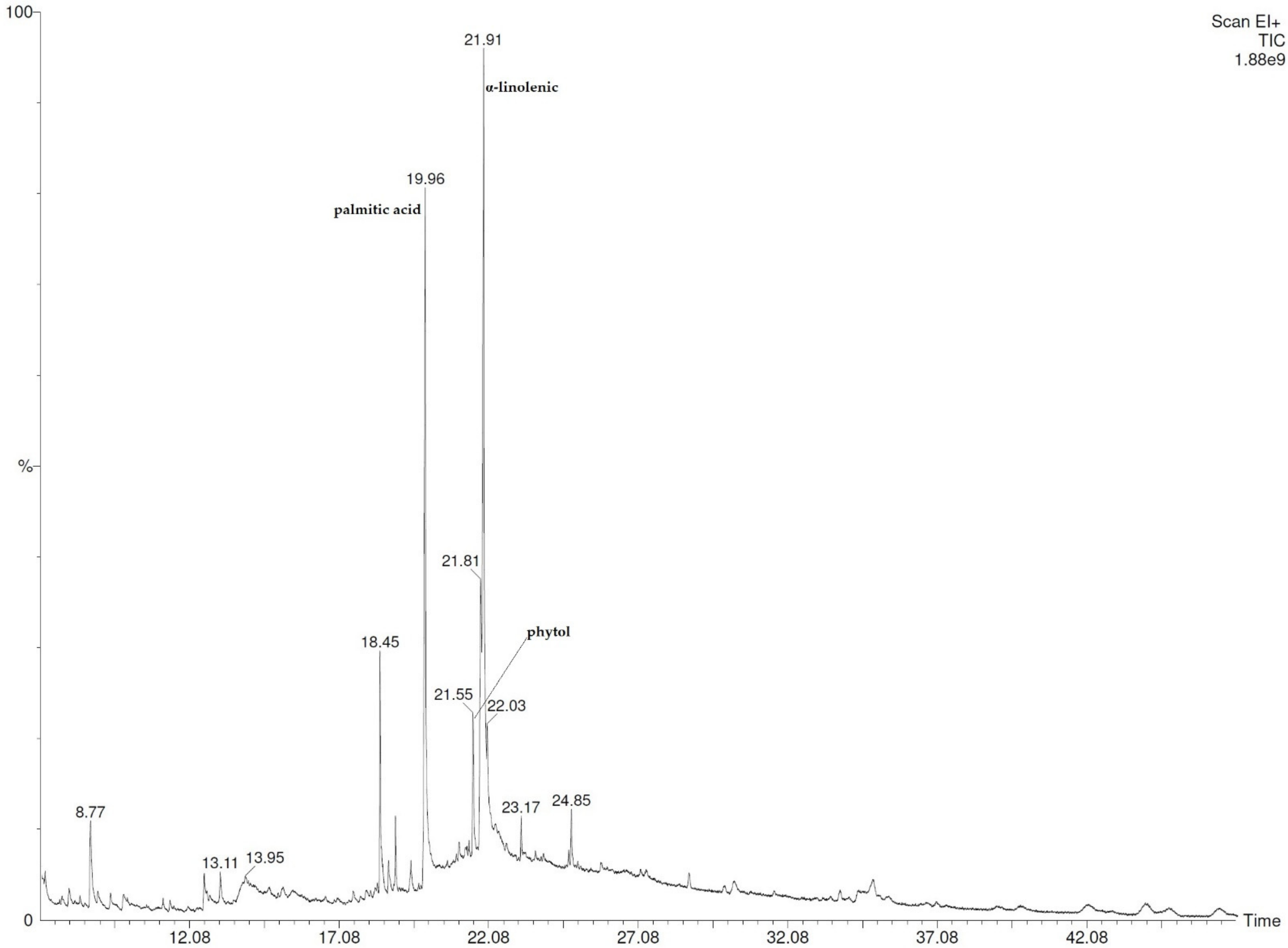

Figure S2. GC/MS Chromatogram of *T. officinale* dried aerial parts after derivatization.

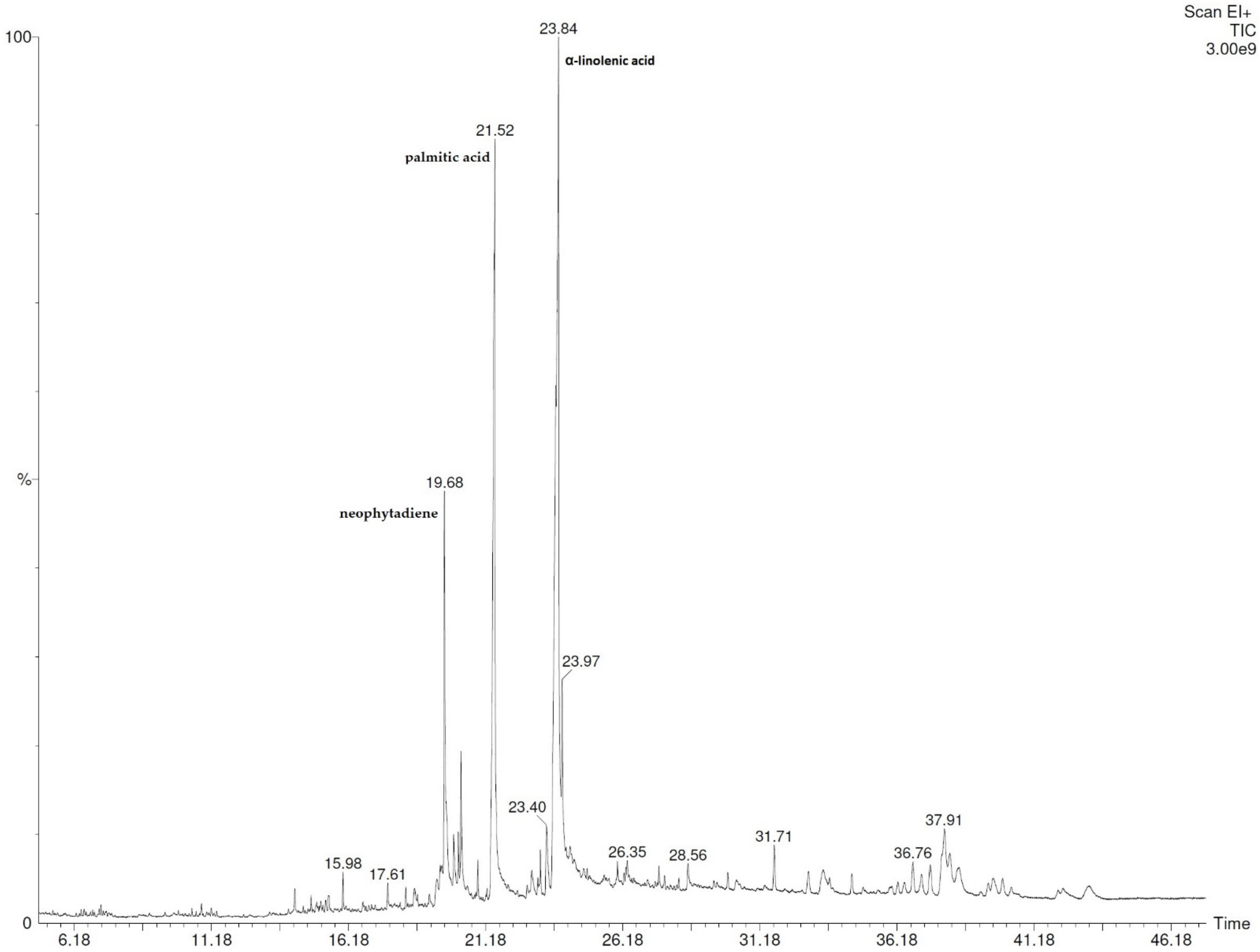

Figure S3. GC/MS Chromatogram of *T. officinale* DCM extract.

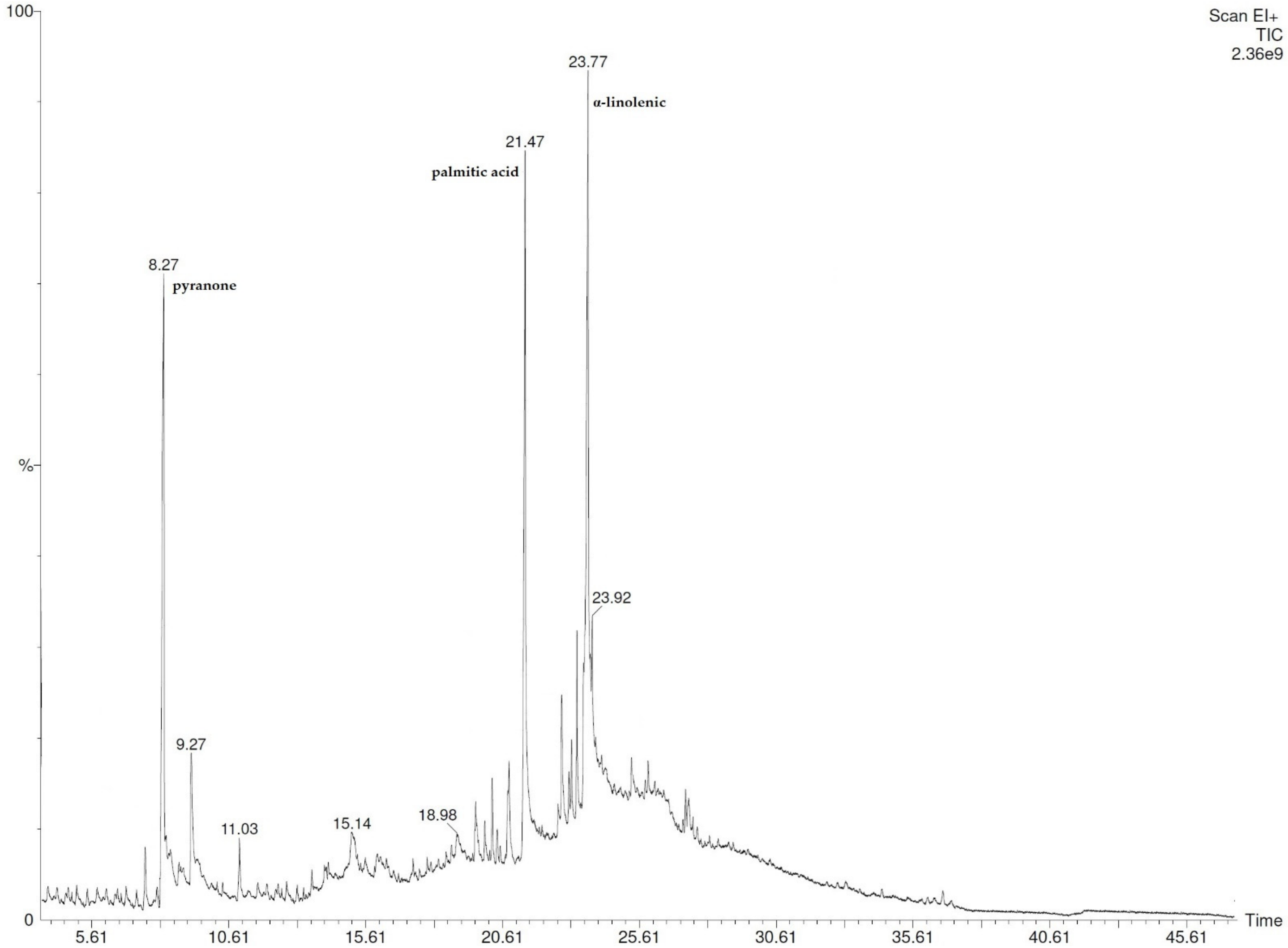

Figure S4. GC/MS Chromatogram of *T. officinale* MeOH extract.

Disclaimer/Publisher's Note: The statements, opinions and data contained in all publications are solely those of the individual author(s) and contributor(s) and not of MDPI and/or the editor(s). MDPI and/or the editor(s) disclaim responsibility for any injury to people or property resulting from any ideas, methods, instructions or products referred to in the content.
